# The impact of parental involvement laws on minors seeking abortion services: a systematic review

**DOI:** 10.1093/haschl/qxad045

**Published:** 2023-09-18

**Authors:** Alisha Kramer, Angeline Ti, Lisa Travis, Adrienne Laboe, Walter O Ochieng, Marisa R Young

**Affiliations:** Department of Gynecology and Obstetrics, Emory University School of Medicine, Atlanta, GA 30322, United States; Family Medicine, Wellstar Hospital, Morrow, GA 30260, United States; Woodruff Health Sciences Center Library, Emory University, Atlanta, GA 30322, United States; Department of Obstetrics and Gynecology, Madigan Army Medical Center, Joint Base Lewis-McChord, WA 98431, United States; EconHealth, LLC, Atlanta, GA 30333, United States; Department of Gynecology and Obstetrics, Emory University School of Medicine, Atlanta, GA 30322, United States

**Keywords:** abortion access, parental involvement laws, parental notification, parental consent, health care for minors, teenage health care, women's health care access

## Abstract

On June 24, 2022, the US Supreme Court overturned the constitutional right to abortion in *Dobbs v Jackson Women's Health Organization*. Minors are a vulnerable population with a high risk of unintended pregnancy who are likely to be disproportionately affected by abortion restrictions. Examining the impact of historical abortion restrictions in minors may provide insight into the anticipated effects of the *Dobbs* decision. This study is a systematic review examining the impact of parental involvement (PI) laws on minors seeking abortion services. Our review suggests an association between PI laws and decreased abortion rates. Parental involvement laws also may increase pregnancy and birth rates and out-of-state travel for abortion procedures and lead to later abortions, although effects appear to be heterogenous.

## Introduction

On June 24, 2022, the US Supreme Court voted 5 to 4 to overturn the constitutional right to abortion in *Dobbs v Jackson Women's Health Organization*. This historic decision will be far reaching in its impact, and vulnerable populations will be disproportionately affected. Minors are a vulnerable population with the highest risk of unintended pregnancy.^1^ These pregnancies are associated with an increased risk of adverse outcomes for both pregnant individuals and children.^2,3^ This study is a systematic review examining the impact of parental involvement (PI) laws on minors seeking abortion services pre-*Dobbs* decision.

In 1979, 6 years after the passing of *Roe v Wade*, the US Supreme Court ruled in *Bellotti v Baird* that states could require a pregnant, unmarried minor to obtain parental consent prior to obtaining an abortion.^4^ Over the following 4 decades, 37 states passed various PI laws.^5^ Parental involvement laws fall into the general categories of need for minors to obtain parental consent to abortion services or need for minors to notify parents of intention to access abortion services.

While minors have legal autonomy to make decisions with regard to contraception and a pregnancy they choose to continue, abortion is a unique aspect of sexual and reproductive health for which they are not allowed independent decision-making capacity. In the absence of PI laws, studies suggest that the majority of minors inform a parent or a guardian of their decision to obtain an abortion.^6^ Minors who choose not to involve their parents commonly cite the desire to preserve the parent–child relationship and maintain autonomy. Others express fear of violence, being forced out of their home, or the inability to involve parents due to logistical barriers.^6,7^ Since the majority of minors seeking abortion services already involve an adult, PI laws may impact the most vulnerable minors who do not feel that they can safely involve a parent/guardian.

Legal abortions are safe and effective, and limiting access to abortion increases the risk of adverse social and health-related outcomes.^8^ Parental involvement laws could result in a number of adverse outcomes, including the following: emotional hardship, risk of violence, delays in care with resultant need for a higher risk procedure at a later gestational age, need for out-of-state travel to obtain abortion services, or inability to access abortion services. Our findings may help anticipate consequences of further limitations to abortion access resulting from the *Dobbs* decision.

## Data and methods

### Search strategy

This systematic review was performed according to recommendations in the Cochrane Handbook and reported in accordance with the Preferred Reporting Items for Systematic reviews and Meta-Analyses (PRISMA) 2009 statement.^9^ A review protocol was developed and registered in the PROSPERO International Prospective Register of Systematic Reviews (registration number CRD42020157863). A systematic literature search strategy was developed with an expert in library sciences (Table S1). We identified English-language publications examining the impact of PI laws on minors seeking abortion services from the passing of *Roe v Wade* in 1973 to 2021, through searches of PubMed, CINAHL, EMBASE, Web of Science, PsycINFO, and Scopus.

**Figure 1.**
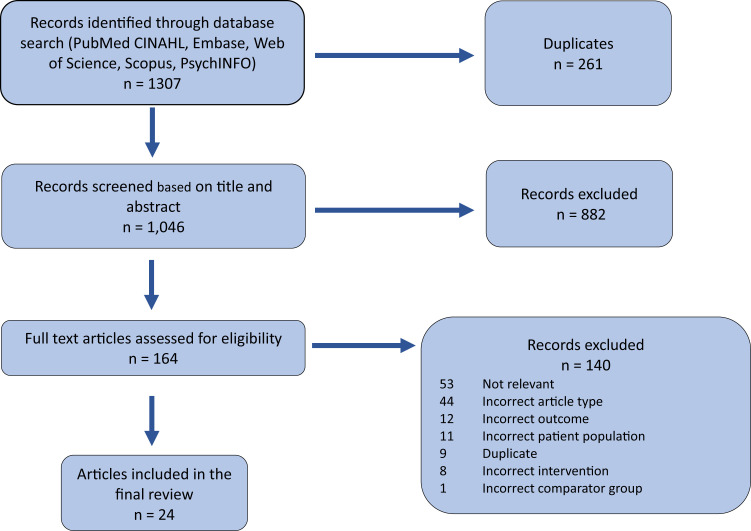
PRISMA (Preferred Reporting Items for Systematic reviews and Meta-Analyses) diagram.

Two authors (A.K. and L.T.) independently screened titles and abstracts for potential inclusion using Covidence (Covidence systematic review software, Veritas Health Innovation, Melbourne, Australia; available at: www.covidence.com). The same reviewers assessed the full texts of the identified articles based on the initial screening. Discrepancies were resolved by a third reviewer (A.T.) (Figure 1).

### Inclusion and exclusion criteria

Studies eligible for inclusion were those published in peer-reviewed journals that examined the impact of PI laws, defined as parental consent or parental notification, on minors in the United States seeking abortion care from 1973 to 2021. Studies examining judicial bypass were excluded. Studies had to disaggregate outcomes data for minors less than 18 years of age specifically. Case reports, commentaries, and review articles were excluded.

### Outcomes

The outcomes of interest included the following: abortion rate, birth rate, pregnancy rate, gestational age at time of abortion, delay in care to receiving abortion services, second-trimester abortion rate, and out-of-state travel to obtain abortion services.

### Data extraction

Two authors (A.K. and A.L.) extracted data from eligible studies using a data-extraction form (key elements reported in Table 1). Abstracted data were discussed among the 3 reviewers (A.K, A.L., and A.T.) and disagreements resolved through consensus.

**Table 1.** Summary of reported outcomes by study.

Study	Risk of bias	State(s)	Law type	Time period	Number of observations	Comparison group	Abortion data source	Statistical methods	Outcomes
Altman-Palm and Tremblay^10^	High	Multi—(33 states in 1989; 41 states in 1992)	NOS	1989–1992	Not given	15–17-y-olds in states without PI laws	CDC, US Census Bureau	Ordinary least-squares regression	Abortion rates: 0.38–0.42 percentage points lower in states with PI laws. Birth rates: Adolescents in states with PI laws have birth rates 3.01% to 3.2% compared to an average of 3.36%. Pregnancy rates: Teen pregnancy rates, 0.57–0.72 percentage points lower in states with PI laws than nationwide mean pregnancy rate. Travel: Abortion rates not significantly affected by teens crossing state lines to avoid PI laws.
Cartoof and Klerman^11^	Low	MA	Consent	1977–1982	210 242 abortions	Pre-law minors	MA Department of Public Health	Univariate time series	Abortion rates: 43% decrease in the monthly average abortion rate in the 20 mo after the MA consent law went into effect. Birth rates: Time-series analysis of births to minor women suggest PI laws may be associated with increased birth rates. Travel: 130% increase in the number of minors traveling outside of MA to obtain abortions services after implementation of the state's consent law, which accounted for the reduction in abortions that occurred after the legislation went into effect.
Colman and Joyce^12^	Low	TX	Notice	1997–2003	2116 abortions	Pre-law minors conceiving at 17 y, 6 mo	Texas Department of State Health Services	Poisson regression model	Abortion timing: Of minors aged 17 y, 8 mo, who delayed until age 18, mean GA at time of abortion was 18.2 wk (all had abortion after 12 wk of GA), as opposed to 8.4 wk among teens of the same age who had their abortion before they turned 18 y. Minors aged 17 y, 9 mo, who delayed until age 18 y had a mean GA at time of abortion of 13.3 wk (50% had abortion after 12 wk of GA), compared to 7.5 wk among those in the same age group who had their abortion before they turned 18 y. Minors aged 17 y, 8–9 mo, experienced a 3% increase in proportion of pregnancies terminated in the second trimester, a relative increase of 22%.
Colman et al^13^	Mod	TX	Notice	1998–2001	Not specified	Pre-law minors aged 17 y	Texas Department of State Health Services	Difference-in-difference model	Birth rates: No significant difference in minors’ birth rates when exposure to the law was based on mother's age at time of conception. Pregnancy rates: No significant change in pregnancy rate among TX minors when exposure to notification law based on mother's age at time of conception.
Ellertson^14^	Mod	MN	Notice	1977–1990	830 697 abortions	Pre-law minors <18 y	MN State Health Departments, US Census Bureau	Poisson regression model, logistic regression model	Abortion rates: In-state abortion rates for minors decreased by 26%. Birth rates: No evidence that PI laws increase birth rates among minors in any of the 3 states. Pregnancy rates: In-state pregnancy rates decreased for minors by 9.1% in MN.
		MO	Consent	1977–1990	533 625 abortions		MO State Health Department, US Census Bureau		Abortion rates: In-state abortion rates for minors decreased by 20%. Birth rates: No evidence that PI laws increase birth rates among minors in any of the 3 states. Pregnancy rates: In-state pregnancy rates decreased 2.3% for minors in MO. Travel: 50% increase in number of minors traveling out-of-state to obtain an abortion after enforcement of MO's consent law.
		IN	Consent	1978–1988	387 237 abortions		IN State Health Department, US Census Bureau		Abortion rates: In-state abortion rates for minors decreased by 17%. Birth rates: No evidence that PI laws increase birth rates among minors in any of the 3 states. Pregnancy rates: In-state pregnancy rates decreased for minors by 10.6% in IN.
Henshaw^15^	Low	MS	Consent	1993	5550 abortions	Pre-law minors <18 y	MS Department of Health	Ratio of the number of abortions obtained by minors to the number obtained by women aged 18 y or older; chi-square test	Abortion rates: 16% decrease in the ratio of abortions obtained by minors to abortions obtained by non-minors for MS residents after implementation of consent law; decrease was offset by a 32% increase in ratio of minors who traveled out of state to obtain abortions. Travel: 32% increase in the ratio of minors to adults seeking abortion services out of state after implementation of MS consent law, which more than accounted for the decreased ratio of minors to adults obtaining in-state abortions.
Joyce and Kaestner^16^	Low	SC, TN	Consent	1987–1991	80 900 abortions	Virginia minors	South Carolina State Vital Registration, Guttmacher Institute	Difference-in-difference-in difference (DDD) estimator to identify the effect of PI law on probability of abortion; time-series plots of the difference in abortion probabilities; ordinary least-squares estimates	Abortion rates: 10.3% decrease in the probability of abortion for non-Black 16-y-olds in SC; otherwise, no significant change in abortion probabilities in SC (consent) or TN (notification) compared to Virginia controls. Birth rates: Birth rates among minors in SC and TN increased with the change in PI laws. Travel: 16-y-olds significantly more likely to travel out of state than 19–20-y-olds to obtain an abortion after implementation of SC's consent law.
Joyce and Kaestner^17^	Low	MS	Consent (2-parent)	1992–1994	23 800 abortions in MS and SC	MS teens younger than 18 y in pre-law period	MS State Vital Registration	Difference-in-difference; Multivariate probit regression; ordinary least-squares regression	Abortion rates: Total abortions among minors (in state and out of state) decreased by 14% compared to a 6% decrease in 19–20-y-olds. In-state abortions in MS decreased by 23% among minors and 3% among 19–20-y-olds. Out-of-state abortions to MS residents increased by 17% among minors but decreased by 15% among 19–20-y-olds. Abortion timing: Associated with a 2.9% increase in the probability of a second-trimester abortion among minors. Travel: Associated with an increase in the number of abortions among minors performed out of state and with an increased probability of an out-of-state abortion among minors relative to non-minors
		SC	Consent (1-parent)	1989–1991		SC teens younger than 17 y in the pre-law period	SC State Vital Registration		Abortion rates: SC's parental consent law did not have a significant effect on abortion rates of minors relative to non-minors. Pregnancy rates: No statistically significant effect of the SC consent law on the timing of abortions among minors. Travel: No significant difference between in-state and out-of-state abortions associated with the SC's consent law
Joyce et al^18^	Low	TX	Notice	1998–2002	394 015 abortions	Texas minors <18 y at time of conception in pre-law period	Texas Department of State Health Services	Relative rate ratios (rate ratio for teens under 18 y divided by rate ratio for 18-y-olds); logistic regression model	Abortion rates: Associated with a decline in abortion rates among all minors, as well as a decrease in the odds that a pregnancy would result in an abortion. Birth rates: Decrease in birth rates among younger minors (under 17 y of age at time of conception) compared to teens who were 18 y old at the time of conception. Subgroup analysis showed that birth rates to non-Hispanic White minors increased significantly, while there was no significant change in birth rate for Hispanic minors or non-Hispanic Black minors. Abortion timing: Increased odds of a second-trimester abortion in a subset of older TX minors aged 17.5 to 17.74 y as compared to 18-y-olds after implementation of a notification law.
Joyce et al^19^	Low	MS, NC, TX	NOS	MS 1985–2013; NC 1985–2013; TX 1985–2013	8920 abortions	Pre-law minors	CDC, Guttmacher Institute	Difference-in-difference; synthetic control	Abortion timing: Out-of-state travel not a significant factor moderating the effect of PI laws.
Kaine and Staiger^20^	Mod	All US states—exact states not specified	NOS	1973–1988, excluding 1983 and 1986 due to data limitations	Unit of observation: county-year, 2524 counties over 14 y	US minors ages 15-17 y in states without parental consent	National Cancer Institute (annual estimate of women residing in a county by race and age), Guttmacher Institute	Weighted least-squares regressions	Birth rates: Increasing distance to an abortion provider associated with fewer minor in-wedlock births while minor out-of-wedlock births remained relatively unaffected by changes in distance to providers in states with PI laws.
Levine^21^	Mod	All US states—exact states not specified	NOS	1985, 1992, 1996, 1999	204 teen-years	Minors in states without PI laws	National Vital Statistics System (birth rates); Guttmacher Institute (abortion data)	Difference-in-difference models, regression models, linear probability models	Abortion rates: 15–20% decrease in abortion rate of minors in states with PI laws. Birth rates: No statistically significant change in births to minors with implementation of PI laws. Pregnancy rates: No statistically significant impact of state PI laws on teen or minor pregnancy rates when adjusting for state-specific trends.
MacAfee et al^22^	High	NH	Notice	2011–2012	373 abortions	Minors <18 y in the pre-law period	Planned Parenthood clinics chart review	*t* Test, chi-square test, Fisher’s exact test, or Wilcoxon rank-sum tests	Abortion rates: Decrease in minors undergoing abortions at Planned Parenthood clinics in NH. Abortion timing: No significant change in number of second-trimester abortions in NH or surrounding states (ME or VT), following implementation of NH's notification law. Travel: Decrease in the number of abortions performed at Planned Parenthood clinics among minors in NH after enforcement of notification law. No increase in the number of minors traveling out of state to seek abortions at Planned Parenthood clinics in VT or ME.
Medoff^23^	Mod	All US states—exact states not specified	NOS	1982, 1992, 2000	Not given	US minors ages 15-17 y in states without PI laws	Guttmacher Institute	Two-stage least-squares regression	Abortion rates: Reduced abortion demand of minors between 46 and 52 abortions per 1000 pregnancies (a decrease of 4.6–5.2% in abortion rate of minors). No significant difference in the impact of notification vs consent laws.
Medoff^24^	Mod	All US states—exact states not specified	NOS	1982, 1992, 2000	Not given	US minors aged 15–17 y in states without PI laws	CDC, Guttmacher Institute	Multivariate Poisson regression	Pregnancy rates: No statistically significant impact of state PI laws on teen or minor pregnancy rates when adjusting for state-specific trends.
New^25^	High	Multi	NOS	1985–2005	Not given	US minors, aged 13–17 years, in states without PI laws	CDC, Guttmacher Institute	Poisson regression with panel-corrected standard errors	Abortion rates: Reduced in-state abortion rate for minors by approximately 15%.
Myers and Ladd^26^	Low	Multi-state—exact states not listed	NOS	1980–2016	3142 counties (county-year panel of measures)	US minors ages 15–18 y in states without PI laws	Individual natality files from NCHS	Double- and triple-difference estimation	Abortion rates: 13.1% reduction in abortions to minors. Birth rates: 1980s: PI laws with 25-mile avoidance distance estimated to decrease teen births by 1.1%. PI laws with avoidance distance of 400 miles estimated to increase teen births by 2.9%, which was not statistically significant. For the later period, a PI law with avoidance distance of 25 miles was estimated to increase the birth rate by 2.3% and avoidance distance of 400 miles was estimated to increase the teen birth rate by 4.6%.
Ohsfeldt and Gohman^27^	Mod	Multi—exact states not listed	NOS	1984–1985, 1988	Not given	Minors <18 y in states without PI laws	MS Department of Health	Linear regression	Abortion rates: 18% decrease in abortion rate. Birth rates: Associated with a 10% increase in minors’ fertility rate. Pregnancy rates: Significant 8% decrease in adolescent pregnancy rates in states with PI laws, but overall, given the 18% decrease in abortion rate, PI laws appear to increase adolescent fertility rates by 10%.
Pierson^28^	Low	MO	Consent	1985–1992	26 357 abortions	Minors <18 y before implementation of consent law	MO Department of Health	No statistical analysis performed	Abortion rates: 41.3% decrease in abortion rates for minors compared to a 23% decline in 18–19-y-olds; decrease was greater among White minors (46% decrease) than among Black minors (19% decrease). Birth rates: Number and percent of pregnancies ending in live births increased after implementation of consent law. Pregnancy rates: Decreasing pregnancy rates in MO more likely due to continuation of pre-existing trend, rather than as result of the legislation. Abortion timing: Trend towards greater proportion of abortions at ≥13 wk of gestation in MO with parental consent law (14% vs 22%) as well as for abortions performed for MO residents out of state (27% vs 35%). Travel: 10% increase in the number of minors leaving MO to obtain abortions after enforcement of the consent law.
Ralph et al^7^	Low	IL	Notice	2012–2014	562 abortions	Minors <18 y at time of conception pre-law	Administrative and medical records at 1 private IL facility	Difference-in-difference; mixed-effects linear and logistic regression models	Abortion rates: 6% decrease in the proportion of abortions obtained by minors relative to non-minor teens after the law went into effect. Abortion timing: Minors traveling to IL from out of state more likely to have a second-trimester abortion in the post-law period, although these results were not statistically significant.
Ramesh et al^29^	Low	IL	Notice	2012–2014	11 816 minors	Minors <18 y obtaining a first-trimester abortion pre-law	Reproductive Health Services Clinic at John H. Stroger, Jr., Hospital of Cook County	Difference-in-difference	Abortion rates: No significant change in number of abortions among minors compared with young adults in IL after enactment of notice law. Abortion timing: No significant difference in GA at time of procedure or in medical vs surgical treatment following implementation of IL notification law.
Rogers et al^30^	High	MN	Notice	1978–1985	1 414 243 minors aged 15–17 y old	Minors aged 15–17 y pre-law	MN Center for Health Statistics	Linear regression model	Abortion rates: Abortion rate of MN minors decreased following enactment of notification law. Birth rates: Birth rates continued to decline after enactment of parental notification law in accordance with long-term trends. Abortion timing: Rate of first-trimester abortions declined among minors more than the rate of second-trimester abortions, thus increasing the ratio of late-to-early abortions among minors.
Tomal^31^	High	AZ, AR, ID, MT, NY, NC, SC, OR, UT, VA, WA	NOS	1995	597 counties	Minors, 15–17 y, in states without PI laws	State county level data, US Census Bureau	Log-linear regression model	Abortion rates: Statistically significant lower abortion rates for both notification and consent laws. Birth rates: Both parental consent and notification laws were associated with significantly higher birth rates for minor and non-minor teens.

Abbreviations: CDC, Centers for Disease Control and Prevention; GA, gestational age; Mod, moderate; NCHS, National Center for Health Statistics; NOS, not otherwise specified; PI, parental involvement.

Parental involvement laws were classified as notification laws or consent laws. In some studies, both consent and notification laws were in place, the type of law was not reported, or data were analyzed from multiple states with different types of laws. In this case, the type of law was classified generically as not otherwise specified (NOS).

**Figure 2.**
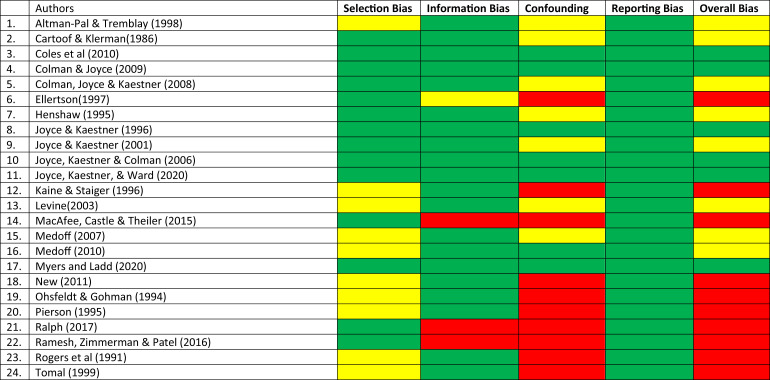
Risk-of-bias analysis. Key: green = low, yellow = moderate, red = high risk of bias.

Risk of bias was assessed using a modified Risk of Bias in Non-randomized Studies of Interventions (ROBINS-I) instrument.^32^ Our structured instrument provided a framework for the assessment of risk of bias in the following domains: selection bias, information bias, confounding, and reporting bias. Selection bias examined the differences between baseline characteristics of our control and comparison groups. Information bias assessed whether study variables were accurately measured. Reporting bias assessed whether the outcomes measured were prespecified and whether all prespecified outcomes were reported. Confounding bias was assessed based on the characteristics of the control group and whether effect estimates were adjusted for state and demographic variation, time trends, and out-of-state travel. Risk of bias was reported as low, moderate, or high (Figure 2).

## Results

Twenty-four studies met the inclusion criteria (Figure 2). Results disaggregated by outcome are discussed below (see also Table 1 with more detailed information).

### Abortion rates

Sixteen of the 24 studies^7,10,11,14,15,18,19,21-23,25-28,30,31^ found decreases in abortion rates in states with enactment of PI laws (4 Consent, 3 Notice, 9 NOS). Of these studies, 7 had a low risk of bias, 4 had a moderate risk of bias, and 5 had a high risk of bias. One low-risk-of-bias study in Illinois found no difference in abortion rates after enactment of a parental notice law.^29^

Finally, 2 low-risk-of-bias studies had mixed results. One found no significant change in abortion rates in South Carolina and Tennessee after enactment of PI laws,^16^ except for the subpopulation of 16-year-olds who were non-Black in South Carolina who were found to have a decreased probability of accessing abortion. The other study^17^ analyzed the effect of Mississippi's 2-parent consent law and South Carolina's 1-parent consent law. In Mississippi, total abortions among minors decreased, whereas in South Carolina, the parental consent law did not have a significant effect on abortion rates of minors.

### Birth rates

Six studies (2 Consent, 4 NOS) found that PI laws were associated with an increase in minors’ birth and fertility rates.^11,16,26-28^ Of these, 4 were low risk, 1 was moderate risk, and 1 was classified as high risk of bias. Four moderate-risk-of-bias studies found no evidence that PI laws impact birth rates among minors (1 Consent, 1 Notice, 2 NOS).^13,14,20,21^ Three studies^10,18,30^ found that PI laws were associated with decreased birth rates to minors (2 Notice, 1 NOS; risk of bias: 1, low; 1, moderate; 1, high), although in 1 case the observed decline was consistent with long-term population-level trends^30^ and less likely as a result of the law.

### Pregnancy rates

Three studies found that states with PI laws have significantly lower pregnancy rates among minors (3 NOS). Of these 3 studies, 2 had moderate risk of bias and 1 had a high risk of bias.^10,14,27^ One study (Consent, low risk of bias) found decreasing pregnancy rates among minors that were likely due to the continuation of a pre-existing trend, rather than as a result of the PI law.^28^ Three studies found no significant change in the pregnancy rate associated with PI laws (1 Notice, 2 NOS; risk of bias: 3 moderate).^13,21,24^

### Abortion timing

Five studies^12,17,18,28,30^ found an increase in second-trimester abortions among minors associated with PI laws (2 Consent, 3 Notice; risk of bias: 4, low; 1, high). One of these studies is highlighted in greater detail below. Five studies^7,14,15,22,29^ found no significant change in the number of second-trimester abortions associated with enactment of PI laws (2 Consent, 3 Notice; risk of bias: 3, low; 1, moderate; 1, high). In 1 of these studies^14^ although there was no difference in second-trimester abortions in Minnesota, a parental notification law was associated with a delay in abortion timing past the eighth week of gestation (*P* < .001) in minors when compared with older women.

One study^17^ had mixed results. Although the authors found no association between South Carolina's consent law and the timing of abortions among minors, they did find that Mississippi's consent law resulted in abortions at later gestational ages among minors relative to non-minors (0.6 wk later; *P* < .05).

Following the implementation of a parental notification law in Texas,^12^ the authors found that pregnant minors who were aged 17 years and 8–9 months at the time of conception were more likely to delay obtaining an abortion until turning 18, resulting in abortions performed at statistically and highly clinically significant later gestational ages (18.2 wk vs 8.4 wk). Overall, there was a 3% increase in the proportion of pregnancies terminated in the second trimester due to delaying abortions in these age groups, which represents an increase in the second-trimester abortion rate of 22% (*P* = .06).

### Resident travel out of state

Six studies, all examining parental consent laws, found an increase in minors traveling out of state for abortion services after implementation of the PI laws.^11,14-17,28^ Of these 6 studies, 5 had a low risk of bias and 1 had a moderate risk of bias.

Three studies^10,19,22^ found no difference in out-of-state abortions after PI laws (1 Notice, 2 NOS; risk of bias: 1, low; 2, high).

## Discussion

In this first systematic review on the effect of PI laws on minors seeking abortion services, we found consistent evidence that PI laws result in decreased rates of abortions to minors aged 17 and under. However, there is heterogeneity in this outcome, as well as heterogeneity of effect across the other outcomes analyzed.

In contrast, a narrative review performed in 2009 by Dennis et al^33^ that examined the impact of PI laws in 29 published studies found that PI laws resulted in an increase in out-of-state travel by minors to obtain abortion services to states that had less restrictive laws but no difference in abortion rates. The authors also concluded that PI laws had little impact on minors’ birth and pregnancy rates.

Parental consent laws are the most restrictive type of PI law, as they require a minor to receive documented consent from either 1 or both parents prior to obtaining an abortion. Parental notification laws require only notification of a parent. While some studies found no difference in outcomes when comparing consent with notification laws, greater impacts were generally seen in states with consent laws. Parental consent laws were associated with decreased abortions in all 5 included studies, while birth rates increased in 2 out of 3 studies that reported on this outcome.

The impact of parental notification laws is less clear. Five out of 6 studies showed a decrease in abortions associated with these notification laws and both studies that reported on delays in accessing abortion care found significant delays. However, the impact of notification laws on the remaining study outcomes suggests either no association or the available studies report conflicting results. Similar to the reasoning above, the lack of consistent association may be explained by the observation that notification laws are less stringent than consent laws in that a minor can, in theory, still obtain an abortion after notifying a parent or guardian.

Multiple studies found that the decreases in abortion rates were offset by minors traveling out of state to obtain abortion services. This out-of-state travel places significant financial and emotional burden on patients seeking care and contributes to widening disparities in care by limiting access to abortion services for the most vulnerable patients. Minors with financial resources and social support may be able to access care, while those without resources may have no alternative but to continue their pregnancy. Some minors may attempt to end the pregnancy on their own through self-managed abortion. Self-managed abortion may be higher among adolescents,^34^ although the prevalence of self-managed abortion, especially in individuals under age 18, who are often excluded from population-based surveys, remains unknown.^35^

Most studies examining the impact of parental consent laws observed an increase in birth rates. This suggests that consent laws do not have a significant impact on sexual activity or contraceptive use. If the goal of PI laws is to decrease sexual activity among minors or decrease pregnancy rates, then other interventions such as comprehensive sexual education and increased access to contraception may be more effective strategies that empower patients, while adhering more closely to the medical ethics principle of patient autonomy.

Given this observed increase in birth rate, it is important to consider the associated increases in pregnancy morbidity and mortality. Legal abortions are safe and effective procedures. Complications from abortion procedures have been documented to occur in less than 1% and not more than 5% of abortions in published studies, with severe complications occurring in no more than a fraction of a percentage of patients.^36^ In comparison, complication rates in pregnancy and childbirth are much higher. In 2021, 1205 pregnant persons died in the United States due to pregnancy causes.^37^ It is estimated that 50–100 pregnant persons experience severe morbidity for every single pregnancy-related death.^38^ Disaggregated morbidity and mortality data for minors are not publicly available. A maternal health crisis already exists in the United States. Increasing birth rates by preventing pregnant individuals from accessing abortion services risks exacerbating already unacceptably high rates of pregnancy morbidity and mortality.

The impact of parental consent laws also shows trends toward later gestational ages at the time of abortion and potential increases in second-trimester abortions. Although abortions are overall low-risk procedures, there is an increased risk of complications with increasing gestational ages.^36^ From an economic standpoint, procedures performed at later gestational ages are more time intensive, require more resources, and are more expensive. For patient safety and efficient utilization of health care resources, minimizing delays to care should be a priority.

### Limitations

A limitation of any systematic review is the quality of included studies. Only 25% of studies included in this review were deemed low risk of bias based on risk-of-bias analysis. The risk-of-bias analysis was limited by not all elements of the Robins-I tool being applicable to the designs of the included studies.

The heterogeneity in the included studies also limited our ability to infer effects of PI laws in different contexts and precluded a meta-analysis. Indeed, effects of PI laws may differ across contexts. Comparison groups in the included studies varied by age, time, and location. The exposure also varied widely, as some PI laws require 2-parent consent with identification requirements or even proof of parenthood, while other laws require notification of 1 parent or allow other relatives or guardians to be notified or provide consent.^5^ Some studies examine data nationally, while others focus on a region or state. As a result, authors use different techniques to control for confounding across distance and time.

An overall lack of population-based data on abortions also limits the ability of the review to draw conclusions. Abortion data in the United States come from 3 primary sources: the Guttmacher Institute, the Centers for Disease Control and Prevention, and state health departments. Each data source has important limitations. Known abortion data limitations include the following: abortions tallied by the state of abortion occurrence rather than according to the patient's state of residence, which limits evaluation of abortion rates and out-of-state travel; lack of data stratified by age, especially for minors; and underreporting and incompleteness of reporting between states (Table 2).

**Table 2.** Summary of 3 major abortion data sources and limitations.

	Guttmacher Institute	CDC	State health departments
Frequency of reporting	Periodic	Annual	Annual
Data collection	Survey of abortion providers	State health departments	Individual-level data
Abortions by state of occurrence vs residence	Occurrence	Occurrence	Occurrence
Disaggregation by age	Not available by age—estimate based on distribution of abortions reported by CDC	Available by single year of age for teenagers aged 15–19 y for majority, but not all, states	Available by single year of age for teenagers aged 15–19 y for majority, but not all, states
Quality	Most widely accepted estimate of number of abortions by state of occurrence	Not all states report to the CDC; total number of abortions 15% lower than that reported by Guttmacher; completeness of reporting varies by state	Collects individual-level data on induced abortions, which are not available through the CDC; completeness of reporting varies by state

Abbreviation: CDC, Centers for Disease Control and Prevention.

Finally, the included studies examined data over many years. Since the impact of PI laws is likely greater when there are no neighboring states to which to travel for out-of-state abortion services, the impact of PI laws has likely increased over time as additional states have enacted PI laws.

Strengths of this review include a rigorous study design, adherence to PRISMA guidelines for systematic reviews, and examination of multiple outcomes potentially affected by PI laws, which allows for a more holistic view of downstream health impacts of policy changes.

## Conclusion

Our review suggests an association between PI laws and decreased abortion rates. Parental involvement laws may also increase pregnancy rates, birth rates, and out-of-state travel for abortion services, and lead to abortions at later gestational ages. Heterogeneity of studies limits inference. Available evidence suggests that parental consent laws may result in greater impacts than notification laws. However, many questions remain unanswered. Parental involvement laws are still in effect in 36 states and will continue to impact minors. The recent overturning of *Roe v Wade* paves the way for further restrictions to abortion access and the potential for ever increasing adverse pregnancy outcomes. Additional studies will be needed to examine the impact of extreme restrictions to abortion access on pregnancy morbidity, mortality, and perinatal outcomes.

## Supplementary Material

qxad045_Supplementary_Data
