# Supplementary material for: The impact of parental involvement laws on minors seeking abortion services: a systematic review
Source: Health Aff Sch. 2023 Sep 18;1(4):qxad045. doi: 10.1093/haschl/qxad045 (PMC10986272; doi:10.1093/haschl/qxad045)
Supplement: qxad045_Supplementary_Data [file qxad045_Supplementary_Data.zip › Supplemental_Table_1 Search strategy.docx]

Table 1. Search Strategy

| Search from 1973 to 2021 in the following databases: PubMed, CINAHL, EMBASE, Web of Science, PsycINFO, and Scopus. |
| --- |
| (abort OR aborts OR aborted OR aborting OR abortion OR abortions OR embryotomy OR embryotomies) AND (minor OR minors OR teenager OR teenagers OR teen OR teens OR preteen OR preteens OR preteenager OR preteenagers OR tween OR tweens OR preteen OR preteens OR adolescent OR adolescents OR adolescence OR youth OR youths OR preadolescent OR preadolescents OR preadolescence OR prepubescent OR prepubescents OR prepubescence) AND (parent OR parents OR parental OR guardian OR guardians) AND (consent OR consents OR consented OR consenting OR involve OR involves OR involved OR involving OR involvement OR involvements OR notify OR notifies OR notified OR notification OR notifications OR permit OR permits OR permitted OR permitting OR permission OR permissions) AND (law OR laws OR legislation OR legislative OR bill OR bills OR statute OR statutes OR require OR requires OR required OR requiring OR requirement OR requirements OR policy OR policies OR restrict OR restricts OR restricted OR restricting OR restriction OR restrictions OR judicial bypass OR access) |
